# 1442. Use of a Spaced-Education Platform to Assess Hand Hygiene Auditor Competency

**DOI:** 10.1093/ofid/ofad500.1279

**Published:** 2023-11-27

**Authors:** Ana M Vaughan-Malloy, Renee Lehane, Mary Wells, Thomas J Sandora

**Affiliations:** Boston Children's Hospital, Boston, Massachusetts; Boston Children's Hospital, Boston, Massachusetts; Boston Children's Hospital, Boston, Massachusetts; Boston Children's Hospital, Boston, Massachusetts

## Abstract

**Background:**

Hand hygiene reliability is a top priority in healthcare. Infection Prevention and Control (IPC) programs often rely on clinical personnel to audit hand hygiene performance. Interrater reliability among auditors is critical to ensuring data validity. We evaluated whether spaced education, which increases long-term knowledge retention, could be integrated into auditor competency assessment.

**Methods:**

113 trained hand hygiene auditors were enrolled in a 13-month online spaced-education platform to assess their mastery of knowledge needed for the role. We delivered 46 unique questions (3 per week, with 2 weeks between repeat delivery of a question) followed by rationale; we retired questions after 3 correct answers. A 10-item survey consisting of 9 Likert-scale questions and one free-text comment was emailed to gauge participant satisfaction with the competency program. Frequencies and percentages were used to summarize Likert responses, and themes were elicited from comments.

**Results:**

From November 2021 to December 2022, a total of 12,120 questions were attempted, and 32 of 113 auditors (28%) completed the competency. Auditors answered 70% of questions correctly on the first attempt and 83% on the final attempt. 30 of 113 auditors (27%) responded to the survey. The majority agreed the number and complexity of questions were appropriate (57% and 67%, respectively). 87% reported the platform easy to navigate, 63% reported the spaced-education model worked, and 77% agreed adequate time was provided for completion. 30% of respondents reported the individual competition as enjoyable. Time and effort required for completion was the most common theme identified among auditor comments; several respondents recommended fewer questions at a narrower spacing interval over a shorter time frame due to competing work demands; 5 individuals expressed a preference for an annual computer-based module.

**Conclusion:**

A spaced education competency program improved hand hygiene auditor knowledge in the short term. Completion rate was low and participants expressed a desire for fewer questions over a shorter time frame. This study offers insight into ways to optimize spaced education as a potential tool for hand hygiene competency assessment.

**Disclosures:**

**Ana M. Vaughan-Malloy, MD, MPH**, Asana: Stocks/Bonds|Aurora: Stocks/Bonds|Ayr Wellness: Stocks/Bonds|Bionano Genomics: Stocks/Bonds|Butterfly Network: Stocks/Bonds|Canopy Growth: Stocks/Bonds|Cresco Labs: Stocks/Bonds|CRISPR Therapeutics: Stocks/Bonds|Cronos Group: Stocks/Bonds|Curaleaf Holdings: Stocks/Bonds|Editas Medicine: Stocks/Bonds|Green Thumb Industries: Stocks/Bonds|High Tide: Stocks/Bonds|Iovance Biotherapeutics: Stocks/Bonds|Jushi Holdings: Stocks/Bonds|Moderna: Stocks/Bonds|Organigram Holdings: Stocks/Bonds|Pacific Biosciences of California: Stocks/Bonds|Personalis: Stocks/Bonds|Pfizer: Stocks/Bonds|SNDL Inc: Stocks/Bonds|Terrascend: Stocks/Bonds|Tilray Brands: Stocks/Bonds|Trulieve: Stocks/Bonds

